# Factors associated with non-use of insecticide-treated bed nets among pregnant women in Zambia

**DOI:** 10.1186/s12936-022-04313-4

**Published:** 2022-10-11

**Authors:** Luwi Mercy Mwangu, Relebogile Mapuroma, Latifat Ibisomi

**Affiliations:** 1 School of Public Health, Faculty of Health Sciences, University of the Witwatersrand, Johannesburg, South Africa; 2 Nigerian Institute of Medical Research, Lagos, Nigeria

**Keywords:** Zambia, Zambian Demographic and Health Survey (ZDHS), Non-use of ITNs, Prevalence, Factors associated with, Pregnant women

## Abstract

**Background:**

Despite the Zambian government’s efforts to ensure 80% use of insecticide-treated nets (ITNs) among pregnant women, ITN use remains critically low. Only 49% of pregnant women reported sleeping under an ITN in 2018 in the country. This study aims to determine the prevalence of, and the factors associated with the non-use of ITNs among pregnant women in Zambia.

**Methods:**

This study was a secondary analysis of the data collected during the 2018 Zambian Demographic and Health Survey. One thousand one hundred and thirty-eight (1 138) pregnant women were included in the study. The prevalence of the non-use of ITNs was computed and univariable and multivariable logistic regression models were fitted to determine the factors associated with the non-use of ITNs in the study population.

**Results:**

The study found that 578 (50.8%) pregnant women reported not using an ITN the night before the survey. The results of the multivariable logistic regression indicated that, primary level education (OR = 2.13, 95% CI 1.23–3.68), ITN per household member (OR = 0.01, 95% CI 0.00–0.02), parity (OR = 0.83, 95% CI 0.70–0.99), moderate malaria prevalence provinces (OR = 0.34, 95% CI 0.23–0.50), high malaria prevalence provinces (OR = 0.26, 95% CI 0.18–0.39) and currently in a union (OR = 0.52, 95% CI 0.30–0.88) were significantly associated with the non-use of ITNs.

**Conclusion:**

This study showed a high prevalence of the non-use of ITNs among pregnant women in Zambia. Factors found to be associated with the non-use of ITNs in the study population are: ITN per household member, parity, education, marital status and malaria prevalence provinces. Addressing the identified factors will require intensification of ITN programming and other malaria preventive measures.

## Background

The World Health Organization (WHO) 2020 global health estimates indicate that malaria contributes significantly to mortality and morbidity in low-income countries [1, 2]. Global malaria infections increased from 228 million to 229 million cases between 2018 and 2019 while fatalities from malaria increased from 405 000 to 409 000 over the same period [1, 2]. In 2018 and 2019, Africa had the highest number of malaria cases globally (213 million and 215 million, respectively) and fatalities from malaria in Africa were 380 000 and 386 000 in 2018 and 2019, respectively [1, 2]. Furthermore, the estimated malaria cases in countries with high malaria transmissions in East and Southern Africa including Zambia, increased from 52.2 million to 59 million between 2018 and 2019 [1, 2].

Malaria remains endemic across all ten provinces of Zambia, and its entire population is at risk of contracting malaria [3, 4]. The risk of malaria is most significant among those living in provinces that usually experience a wetter climate, are rural or are impoverished [3]. The 2020 World malaria report showed that Zambia experienced an increase in reported malaria cases from 4 to 824 to 5 068 876 between 2018 and 2019 while the number of malaria deaths increased from 1 209 to 1 339 [2]. The general incidence of malaria in Zambia was 386 per 1 000 persons in 2013, 409 per 1 000 persons in 2014, and 335 per 1 000 persons in 2015 [5].

Malaria disproportionately affects pregnant women. Thus, malaria in pregnancy (MiP) is a significant public health problem, especially, in areas of sub-Saharan Africa that experience moderate to high transmissions of malaria [1, 2]. In 2018, 29% (11.2 million) of pregnant women had malaria infections and this increased to 11.6 million in 2019 [2]. As a result of malaria infections during pregnancy, 872 000 children were born with low birth weight in 2019 [2].

In Zambia, laboratory-confirmed malaria cases increased from 45 to 1 000 pregnant women in 2013 to 64 per 1 000 pregnant women in 2015, with Luapula province, a province with a high malaria prevalence, recording the highest incidence rate of 177 per 1 000 pregnant women in 2015 [5].

Malaria in pregnancy is one of the most preventable causes of negative birth outcomes [6, 7]. The negative outcomes include low birth weight, fetal loss, retarded growth, and premature birth [6, 7]. In malaria-endemic countries, malaria is a major cause of maternal anaemia, which has been associated with obstetric haemorrhage and maternal mortality [1]. Malaria-endemic countries are also disproportionately affected by iron deficiency anaemia, helminthic infections and the human immunodeficiency virus, which exacerbate clinical outcomes of malaria infection in pregnancy [1].

ITNs are an essential preventive intervention that have been proven to reduce the incidence and consequences of MiP [8–13]. Several studies have found that pregnant women who sleep under an ITN have lower odds of contracting malaria when compared to those who do not sleep under an ITN [9–11]. Even when other associated factors such as educational status, parity, number of antenatal visits, number of intermittent preventive treatment doses in pregnancy and age are adjusted for, the odds of contracting malaria remain higher among pregnant women who do not sleep under an ITN [9–11]. Studies have also shown that sleeping under an ITN is beneficial to the health of pregnant women and their unborn children [11, 12, 14]. For instance, in Kenya using an ITN during pregnancy reduced the incidence of severe maternal anaemia by 47%, low birth weight by 28% and maternal malaria parasitemia by 38% [14]. A systematic review of studies conducted in Africa showed that the use of ITNs decreased low birth weight by 23% and reduced fetal loss by 33% [12]. The findings were similar in a meta-analysis that assessed the efficacy of intermittent preventive treatment in pregnancy (IPTp) and the use of ITNs in pregnancy in 25 African countries. The use of IPTp or an ITN during pregnancy were found to reduce the incidence of neonatal mortality and low birth weight in newborns by 82% and 79.2%, respectively in the meta-analysis [8]. They concluded that the use of the two preventive interventions help reduce the incidence of MiP [8].

In areas where malaria is endemic, including Zambia, the WHO recommends the use of ITNs [2]. Zambia put in place a policy for mass distribution of ITN in 2005 [15]. The policy entails routinely distributing ITNs to pregnant women and under-five children [16]. However, despite the Zambian government’s efforts to ensure continual distribution of ITNs to pregnant women, ITN use among pregnant women remains critically below the 80% target set in 2011 [17].

Estimates from the 2018 Zambian Demographic and Health Survey (ZDHS) indicate that 51% of pregnant women did not sleep under an ITN the night before the survey, putting them at risk of contracting malaria. Therefore, determining the factors associated with the non-use of ITNs among pregnant women in Zambia is important for unblocking barriers to ITN use thereby, preventing malaria in this group of women.

## Methods

### Study setting and design

Zambia is a country in Southern Africa that experiences seasonal malaria transmission [4]. The rainy season (December to April) gives rise to malaria transmission peaks between January and April and the malaria parasite prevalence peak is towards the end of the transmission period (April and May) [4]. This study is a secondary analysis of the population-based cross-sectional data collected during the 2018 ZDHS. The ZDHS occurred between 18 and 2018 and 24 January 2019, which was outside the malaria transmission and parasite prevalence peak periods. Details of the methodology used during the ZDHS are published in the ZDHS report [18]. Thirteen thousand, six hundred and eighty-three women aged 15–49 years were interviewed in the survey. Of these women, 1138 women were pregnant at the time of the survey and they form the analysis sample for this study.

### Outcome variable

The outcome variable is binary, which was the use or non-use of an ITN by a pregnant woman. Non-use of an ITN was defined as not sleeping under an ITN the night before the survey. If a pregnant woman slept under an ITN the night before the survey, the code assigned was zero (0) and if a pregnant woman did not sleep under an ITN the night before the survey, the code assigned was one (1).

### Explanatory variables

The explanatory variables are age, employment status, ITN per household member, place of residence, parity, educational attainment, religion, marital status, wealth index and province. The literature reviewed aided in the variable selection [19–30]. A detailed description of the variables, codes, and how they are operationalized in this study is presented in Table 1.

**Table 1:** Description of variables, codes, and reclassifications

Explanatory variables	Code in the original data set	How the variable is coded/used in this study
ITN per household member	N/A	ITN per household member is a derived variable calculated by dividing the number of ITNs in households by number of de facto household members. Continuous (0–2) and Categorical. 0 = 0; No net 1– 0.49 = 1; Not sufficient 0.5 = 2; Sufficient > 0.5 = 3; More than sufficient
Age	Continuous	Continuous (15–49) and categorical 15–24 = 1; 25–34 = 2; ≥35 = 3
Parity	Continuous	Continuous (0–12) and categorical 0 = 0; 1 = 1; 2–4 = 2; ≥5 = 3 [32, 33]
Place of residence	Urban = 1; Rural = 2	Urban = 1; Rural = 2
Educational attainment	No education = 0; Primary = 1; Secondary = 2; Higher = 3	No education = 0; Primary = 1; Secondary = 2; Tertiary = 3
Religion	Catholic = 1; Muslim = 2; Protestant = 3; Other = 96	Muslim and Other = 0; Catholic and Protestant = Christians = 1
Wealth index	Lowest = 1; Second = 2; Middle = 3; Fourth = 4; Highest = 5	Poor (1 & 2) = 1; Middle (3) = 2; Rich (4 & 5) = 3
Employment status	Not working = 0; Professional/technical/managerial = 1; Clerical = 2; Sales = 3; Agricultural or self-employed = 4; Household and domestic = 5; Services = 6; Skilled manual = 7; Unskilled manual = 8	0 = 0 = Unemployed and 1–8 = 1 = Employed
Marital status	Never in union = 0; Currently in union and living with a man = 1; Formerly in union and formerly lived with a man = 2	Never in union = 0; Currently in union and living with a man = 1 = Currently in union; Formerly in union and formally lived with a man = 2 = Formerly in union
Provinces renamed to provincial malaria categories	Central = 1; Copperbelt = 2; Eastern = 3; Luapula = 4; Lusaka = 5; Muchinga = 6; Northern = 7; North-Western = 8; Southern = 9; Western = 10	Categorized accorded to malaria parasite prevalence [3, 34] Low malaria prevalence provinces (1, 5 & 9) = 0; Moderate malaria prevalence provinces (2, 3 & 10) = 1; High malaria prevalence (4, 6, 7 & 8) = 2

### Statistical analysis

This study’s analysis used sampling weights and survey estimation commands to account for the stratification and clustering in the ZDHS data. For this study, all statistical tests were done at alpha 0.05 and 95% confidence intervals. The software used was STATA SE version 16 (Statacorp LP, College Station, Texas, USA).

To determine the prevalence of the non-use of ITNs among pregnant women aged 15–49 years in Zambia, cross-tabulation of the outcome variable by each of the identified explanatory variables was performed. For this part of the analysis, continuous variables (ITN per household member, age and parity), were categorized. The categorization was done to get the prevalence based on the assigned categories. Details of the categorizations are provided in Table 1.

To determine the factors associated with the non-use of ITNs among pregnant women, univariable and multivariable logistic regression models were fitted. The univariable regressions computed the unadjusted odds ratios, while the multivariable regression computed the adjusted odds ratios. To prevent the loss of power of the explanatory variables and to prevent inflating the type-I error rate [31], continuous variables (ITN per household member, age and parity) were not categorized for this part of the analysis. The variance inflation factor (VIF) was used to check for multi-colinearity. There was no evidence of multi-colinearity as all VIF values were < 10. The religion variable was not entered into the models because those who were Muslims and from other religions other than Christianity, had very few observations, which caused imprecise predictions.

## Results

### Prevalence of the non-use of ITNs among pregnant women

Table 2 shows the prevalence of non-use of ITNs among pregnant women in Zambia. Overall, 50.8% of the pregnant women did not use an ITN the night before the survey with 43.6% (252 of 578) of the women not having an ITN in their household and only 16.6% (96 of 578) having sufficient/more than sufficient ITNs. The prevalence of women who did not use an ITN decreased as the ITN per household member increased. The result is similar for age and parity of the pregnant women where prevalence of non-use decreases with increase in age and parity. Table 2 further shows that the prevalence of non-use of ITNs was high among pregnant women who have some level of education, live in urban area, are employed, are Christians, are non-poor, are from low malaria prevalence provinces and pregnant women who were never in union.

**Table 2:** Prevalence of the non-use of ITNs among pregnant women

Variable	ITN use		
	No n (%) 578 (50.8)	Yes n (%) 560 (49.2)	Pearson’s Chi -square test p-value
ITN per household member			
No net	252 (100)	−	
Not sufficient	230 (45.8)	272 (54.2)	< 0.0001
Sufficient	45 (24.7)	139 (75.3)	
More than enough	51 (25.6)	149 (74.4)	
Age			
15–24	312 (57.7)	229 (42.3)	
25–34	191 (45.6)	228 (54.4)	
≥ 35	76 (42.5)	102 (57.5)	0.0093
Parity			
0	149 (60.5)	98 (39.5)	
1	137 (50.2)	136 (49.8)	
2–4	219 (49.9)	220 (50.1)	
≥ 5	73 (50.8)	106 (59.2)	0.0232
Educational attainment			
No education	34 (35.1)	64 (64.9)	
Primary	302 (54.7)	250 (45.3)	
Secondary and tertiary	243 (49.7)	246 (50.3)	0.0044
Place of residence			
Rural	348 (47.8)	380 (52.2)	
Urban	231 (56.1)	180 (43.9)	0.1605
Employment status			
Employed	290 (51.9)	269 (48.1)	
Unemployed	289 (49.8)	291 (50.2)	0.7166
Religion			
Christian	573 (51.5)	539 (48.5)	
Muslim and other	5 (20.4)	20 (79.6)	0.0098
Wealth index			
Poor	232 (45.7)	275 (54.3)	
Middle	126 (57.6)	93 (42.4)	
Rich	221 (53.5)	192 (46.5)	0.0661
Provincial malaria categories			
Low malaria prevalence provinces	306 (67.6)	147 (32.4)	
Moderate malaria prevalence provinces	158 (42.7)	212 (57.3)	
High malaria prevalence provinces	115 (36.4)	201 (63.6)	< 0.0001
Marital status			
Never in union	115 (67.1)	56 (32.9)	
Currently in union	416 (46.7)	474 (53.3)	
Formerly in union	48 (62.3)	29 (37.7)	0.0001

Key: n = total number of pregnant women (survey weighted), % = survey weighted percentage

### Factors associated with the non-use of ITNs among pregnant women

The second column of Table 3 presents the unadjusted odds ratios of the univariable logistic regressions analysis. The results indicate that ITN per household member, age, parity, educational attainment (primary, and secondary), wealth index (non-poor), provincial malaria categories (moderate to high), marital status (currently in a union) were significantly associated with the non-use of ITNs among pregnant women (*p* < 0.05).

**Table 3:** Unadjusted and adjusted odds ratios of the association between the explanatory variables and non-use of ITNs

Variable	Unadjusted odds ratio (95% CI)	Adjusted odds ratio (95% CI)
ITN per household member	0.01 (0.00–0.03)*	0.01 (0.00–0.02)*
Age	0.95 (0.94–0.98)*	1.00 (0.94–1.07)
Parity	0.88 (0.83–0.95)*	0.83 (0.70–0.99)*
Educational attainment		
No education	1.00	1.00
Primary	2.23 (1.33–3.74)*	2.13 (1.23–3.68)*
Secondary and tertiary	1.83 (1.09–3.06)*	1.14 (0.58–2.21)
Place of residence		
Urban	1.00	1.00
Rural	0.72 (0.49–1.04)	0.87 (0.54–1.42)
Employment status		
Unemployed	1.00	1.00
Employed	1.09 (0.78–1.52)	1.17 (0.85–1.62)
Wealth index		
Poor	1.00	1.00
Middle	1.61 (1.00–2.58)*	1.32 (0.85–2.04)
Rich	1.39 (1.00–3.06)*	1.17 (0.69–1.98)
Provincial malaria categories		
Low malaria prevalence provinces	1.00	1.00
Moderate malaria prevalence provinces	0.36 (0.25–0.52)*	0.34 (0.23–0.50)*
High malaria prevalence provinces	0.27 (0.19–0.40)*	0.26 (0.18–0.39)*
Marital status		
Never in union	1.00	1.00
Currently in union	0.42 (0.29–0.64)*	0.52 (0.30–0.88)*
Formerly in union	0.81 (0.41–1.60)	0.99 (0.39–2.53)

Key: ^*^p-values < 0.05, 95% CI = 95% confidence interval

The third column of Table 3 shows the adjusted odds ratios computed from the multivariable logistic regression. In this model, ITN per household member, parity, attainment of primary education, not being from a low malaria prevalence province and currently in a union were statistically associated with the non-use of ITNs among pregnant women (*p* < 0.05).

The odds of not using an ITN decreased as the ITN per household member increased just as the odds of not using decreases with every additional birth. With respect to educational attainment, the odds of not using an ITN among pregnant women who had a primary level of education was 2.13 times the odds of not using an ITN among pregnant women who had no education (95% CI: 1.23–3.68). Furthermore, the odds of not using an ITN among pregnant women from moderate malaria prevalence provinces was 0.34 (95% CI: 0.23–0.50) times less likely than among pregnant women from low malaria prevalence provinces and 0.26 (95% CI: 0.18–0.39) times less likely among pregnant women from high malaria prevalence provinces compared to those from low malaria prevalence provinces.

Regarding marital status, the odds of not using an ITN among pregnant women who were currently in a union were 0.52 (95% CI: 0.30–0.88) times the odds of not using an ITN among pregnant women who had never been in a union. However, age, secondary education, and the household wealth index lost the significance they had in the univariable analysis, although the direction of association remains the same.

## Discussion

This study aimed to determine the prevalence of, and the factors associated with the non-use of ITNs among pregnant women in Zambia. The results show that 50.8% of the women did not use an ITN the night before the survey. This is higher than what was reported in the 2018 Zambia Malaria Indicator Survey report [34]. The high percentage of non-use is explained by the fact that 43.6% of the pregnant women that did not use an ITN the night before the survey did not have any ITN in their household. The high prevalence may also be explained by the fact that the period during which the ZDHS occurred (18 July 2018–24 January 2019) was mainly outside the malaria transmission peak period (January–April) and the peak parasite prevalence period (April–May) [18]. Therefore, the timing of the ZDHS may not have allowed for better compliance to use ITNs. Other studies have noted that ITN use could be seasonal and influenced by factors such as temperature, rain and the density of mosquitoes [35–39].

As per the criterion of 0.5 ITN per household member being sufficient (one ITN for every two persons at risk of malaria) [2, 40], this study showed that only 16.6% of the pregnant women had a sufficient number of ITNs in their household. This could have also contributed to the lower odds of using an ITN [22]. Verifiably, this study found that the odds of not using an ITN decrease as the ITN per household member ratio increases. The finding is plausible because owning sufficient ITNs in the household may ensure that every household member, including pregnant women, sleep under an ITN [22, 41]. This is consistent with studies done in Mali [22] and Malawi [30]. Additionally, it was found that as parity increases, the odds of not using an ITN decreases. This finding is similar to studies done in Uganda [21] and Malawi [30]. The finding may be because pregnant women with more deliveries are more aware of the risks of malaria and the importance of ITN use [21].

This study found that those with some level of education, especially, primary level had higher odds of not using an ITN compared to those with no education. This differs from what was found in studies done in Nigeria, Congo, and Uganda [20, 21, 26], that showed that individuals with no education were less likely to use an ITN. Although, not significant, the non-poor pregnant women also had higher odds of not using an ITN. The education and wealth findings may be because the women in these categories were using other malaria preventative measures such as intermittent preventive treatment in pregnancy (IPTp) or living in better vector-controlled environment.

Further, pregnant women in low malaria prevalence provinces were less likely to use an ITN. This finding is in line with several studies that point out the regional variability of ITN use [30, 42, 43]. Unlike the low transmission provinces, the moderate to high malaria prevalence provinces are the wetter provinces of Zambia [3], with more persistent vector breeding pools and higher mosquito densities. This may explain why pregnant women from these provinces were more likely to use an ITN compared to women from the low malaria prevalence provinces.

This study found that marital status was associated with the non-use of ITNs among pregnant women. The result is in line with two studies done in Kenya and Congo that found that pregnant women in a union are more likely to use an ITN [24, 44]. The findings indicate that pregnant women who had never been in a union are less likely to use an ITN. A possible explanation is that pregnant women in union may be influenced by their partner’s decision to practise malaria prevention behaviours [45]. Additionally, women that had never been in a union tend to be younger, less likely to have had a prior pregnancy and prior exposure to information about the risks of malaria or the importance of ITN use during pregnancy [21, 23].

### Strengths of the study

This study used a nationally representative sample of pregnant women. Therefore, the study findings may be generalized to pregnant women in Zambia. Additionally, the study was able to account for the complex nature of the ZDHS sample design by using survey estimation commands in STATA. Thereby, improving the accuracy of the study estimates.

### Study limitations

The non-use of ITNs the night before the ZDHS survey might have been overestimated or underestimated in this study, because (1) it was self-reported in the survey and may therefore suffer from social desirability bias, and (2) the 2018 ZDHS occurred outside the malaria transmission peak period in Zambia. Hence, some of the pregnant women may be users of ITNs during other times of the year. In addition, not all the variables that needed to be adjusted for were included in the analysis. For instance, the 2018 ZDHS did not collect information on the knowledge and beliefs about malaria, seasonality of malaria and weather conditions in Zambia, which have been shown to influence ITN utilization [20, 26, 29, 46]. Further, religion, which has been shown to influence ITN use in other settings [24, 47] was not used in the models due to the variable having very few observations for the Muslim/Other category.

### Policy implications and recommendations

The study shows a high prevalence of the non-use of ITNs among pregnant women in Zambia with over two-fifths of the non-users having no ITN and only 16.6% having a sufficient number of ITNs in their household. This shows a need to expand ITN distribution programming to reach all the pregnant women that need them. Further, although the survey was conducted in malaria off season period in the country, it is important to increase and sustain the use of ITN during pregnancy as the risk of contracting malaria among the group is not totally absent.

The factors found to be associated with the non-use of ITNs in this study suggest areas that need to be targeted for optimizing malaria prevention efforts. Finding less likelihood of not using an ITN with increase in number of ITN per household member also points to the need to increase ITN supply to households. The ITN distribution systems need to consider the number of household members to ensure that households own sufficient ITNs.

This study found a decline in non-use with increase in parity and higher odds of non-use among pregnant women that were never in a union. There is a need to have targeted and intensified ITN programming among this group as they tend to be young and may not have prior exposure to pregnancy or information about the risks of malaria or the importance of ITN use during pregnancy.

The finding of higher odds of using ITNs in the moderate to high malaria prevalence provinces, suggests that the women are aware of the dangers of contracting malaria and the importance of using ITN to protect themselves and their unborn children. Thus, intensifying ITN programming in all parts of Zambia, especially in the low malaria prevalence provinces that have shown less likelihood of use is paramount.

The less likelihood of using an ITN by pregnant women with some level of education in this study is concerning as they constitute 94% of the non-users (545 of 578 – see Table 2). However, they may be using other preventive interventions. It is thus, imperative to intensify ITN messaging as well as promote other preventive measures among pregnant women in Zambia.

The high number of pregnant women with no ITN in their household is alarming. Research on the prevalence of ITN ownership and the factors associated with it is recommended to start addressing this concerning issue. Future research on the non-use of ITNs among pregnant women in Zambia should consider exploring why women with some level of education and non-poor women were less likely to use an ITN. Further, adjusting for variables such as knowledge, beliefs, and practices about malaria may provide further insight into why pregnant women in Zambia are using or not using ITNs.

In terms of monitoring and evaluation, the factors found to be associated can be used as indicators that track the progress of malaria prevention interventions among pregnant women in Zambia. The continual monitoring of progress will ensure the unblocking of barriers to ITN use thereby, preventing malaria in this group of women.

## Conclusion

This study showed a high prevalence of the non-use of ITNs among pregnant women in Zambia. Factors found to be associated with the non-use of ITNs in the study population are: ITN per household member, parity, education, marital status and malaria prevalence provinces. Addressing the identified factors will require intensification of ITN programming and other malaria preventive measures.

## Data Availability

The dataset analysed for the current study is available on Demographic and Health Surveys Programme website, https://dhsprogram.com/data/available-datasets.cfm.

## Abbreviations

ITN: Insecticide-treated net
WHO: World Health Organization
ZDHS: Zambian Demographic and Health Survey
MiP: Malaria in pregnancy
